# Risk Factor Analysis for Vascular Occlusions After Dermal Filler Injections: A Systematic Review and Meta-Analysis

**DOI:** 10.7759/cureus.82800

**Published:** 2025-04-22

**Authors:** Adham Chakhachiro, Maaz Waseem

**Affiliations:** 1 Dermatology, Clinic Platina, London, GBR; 2 Internal Medicine, St. Peter's Hospital, Chertsey, GBR; 3 Biological Sciences, Atta-Ur-Rehman School of Applied Biosciences, National University of Sciences and Technology (NUST), Islamabad, PAK

**Keywords:** hyaluronic acid fillers, hyaluronidase, meta-analysis, reconstructive dentistry, retinal vascular occlusions

## Abstract

This systematic review and meta-analysis investigate the risk factors associated with vascular occlusions following dermal filler injections, a rare but serious complication in aesthetic medicine. Fourteen studies involving various filler materials and injection sites were analyzed to identify patient and procedural variables influencing clinical outcomes. The most frequently used filler was hyaluronic acid (HA) (61.3%), and most complications occurred in female patients (71%). Anatomical regions with complex vasculature, such as the glabella, nose, and nasolabial folds, were most commonly implicated. Recovery outcomes were significantly influenced by the specific vessels involved, with occlusions in smaller arteries showing a better prognosis. Timely recognition and intervention were associated with improved recovery, with delays beyond five days correlating with permanent deficits. Hyaluronidase use in HA-related occlusions yielded high partial or total recovery rates with an 84.2% success rate, reaffirming its role as a first-line treatment. Based on the analysis, a morbidity risk assessment tool was developed to stratify patients into low-, moderate-, and high-risk categories, offering practical value for clinical decision-making. The findings underscore the importance of anatomical precision, prompt intervention, and standardized treatment protocols.

## Introduction and background

Dermal filler injections have become one of the most widely sought-after aesthetic procedures globally in the field of cosmetic dermatology in recent years [1]. With their ability to restore volume, enhance facial contours, and diminish the appearance of wrinkles, these minimally invasive treatments have gained immense popularity [2]. Various filler materials, including hyaluronic acid (HA), calcium hydroxylapatite (CaHa), and poly-L-lactic acid (PLLA), offer tailored solutions to meet the specific needs of diverse patient populations [3]. Despite their widespread acceptance and generally favorable safety profile, dermal fillers are not without risks. Among the most serious complications is vascular occlusion, a rare (0.05-0.01% incidence) but potentially devastating event that can lead to tissue ischemia, necrosis, or even permanent vision loss and stroke [4].

Understanding the risk factors contributing to vascular occlusion is crucial for mitigating this complication and improving patient outcomes [5]. Vascular occlusion occurs when the filler inadvertently enters a blood vessel or exerts external pressure on it, impeding blood flow or causing vasospasm [6]. The consequences range from mild to severe, depending on the site and extent of occlusion. Key risk factors hypothesized in existing literature include the injection technique, the anatomical site of administration, the type of filler used, and the patient's vascular anatomy [7]. However, much of this evidence remains anecdotal or derived from small-scale case studies, leaving significant gaps in our understanding.

According to market trends, the demand for dermal fillers has steadily increased, driven by advancements in techniques, rising aesthetic awareness, and a broader acceptance of cosmetic interventions across various demographic groups [8]. This surge also shows the importance of proper practitioner training and patient education. A deeper understanding of the risk factors associated with vascular occlusions can provide valuable insights into optimizing procedural protocols and refining injection techniques [9].

Previous studies exploring vascular occlusions have predominantly focused on case reports or case series, offering limited generalizability due to small sample sizes and inconsistent methodologies [10]. While these reports have provided valuable initial observations, the lack of robust, aggregated data limits their applicability to clinical practice. Systematic reviews and meta-analyses can address these limitations by synthesizing evidence across multiple studies, offering a comprehensive and statistically robust assessment of risk factors.

This study aims to systematically review and analyze the existing literature on vascular occlusions resulting from all types of dermal filler injections. Specifically, it seeks to identify and quantify the risk factors associated with this complication, focusing on patient demographics, anatomical sites of injection, injection techniques, filler materials, and vessel involvement. By pooling data from a diverse range of studies, this research intends to generate actionable insights that can guide clinical decision-making, enhance procedural safety, and improve patient outcomes. The importance of this study extends beyond its clinical implications. As the cosmetic industry continues to evolve, regulatory agencies and professional organizations are increasingly emphasizing the need for evidence-based practices. By elucidating the risk factors for vascular occlusion, this systematic review and meta-analysis will contribute to the growing body of literature aimed at standardizing safety protocols and setting benchmarks for best practices in aesthetic medicine.

## Review

Methods

Eligibility Criteria

Studies were included if they reported confirmed cases of vascular occlusions following dermal filler injections, provided data on patient demographics, anatomical injection sites, injection techniques, and filler types, and used clear diagnostic criteria for vascular occlusions. Case-control studies, cohort studies, and case series with detailed patient and procedural data were considered eligible. Studies were excluded if they were non-English without translation, lacked sufficient data on variables of interest, or focused on unrelated complications.

Search Strategy

A comprehensive literature search was conducted in PubMed, Embase, and the Cochrane Library using keywords such as “vascular occlusion,” “dermal fillers,” “risk factors,” and “injectable complications.” Boolean operators and database-specific filters were applied to ensure thorough coverage. The search strategy was tailored to capture relevant case-control studies, cohort studies, and case series, ensuring retrospective and prospective data inclusion. The proper Boolean search strings are shown in Table 7 of Appendices.

Study Selection

After removing duplicates, studies were screened in two stages: title/abstract screening and full-text review. Two independent reviewers assessed each study against the inclusion and exclusion criteria. Discrepancies were resolved through discussion or consultation with a third reviewer. Selected studies underwent data extraction and quality assessment to ensure reliability and relevance for the meta-analysis.

Risk of Bias Assessment

The risk of bias and overall quality of the included studies were evaluated using the Joanna Briggs Institute Checklist for Case Reports [11] and summarized. PRISMA guidelines were adhered to throughout the review process to ensure transparency and rigor.

Data Extraction

Data for the meta-analysis, pertaining to study outcomes with relevant study characteristics, were extracted and entered into Microsoft Excel® (Microsoft, Redmond, WA). The recorded data included study authors, patient demographics, age, gender, filler material, as well as injection site, along with relevant medical histories, vessels involved, and status of recovery. The outcome for recovery was categorized into “no improvement” or “partial/total improvement.”

Outcomes and Analysis

Descriptive statistics were employed for all variables. The primary outcome was the status of recovery and the association of various factors with it. The odds ratio (OR) was calculated using Fisher’s exact test, along with associated pooled effects. Significance testing was two-sided, and p<0.05 was considered significant. The analysis was carried out using R Programming Language® with its “metafor” package (ref.) and SPSS version 27® (IBM SPSS Statistics, Armonk, NY).

Results

Study Characteristics

**Figure 1:**
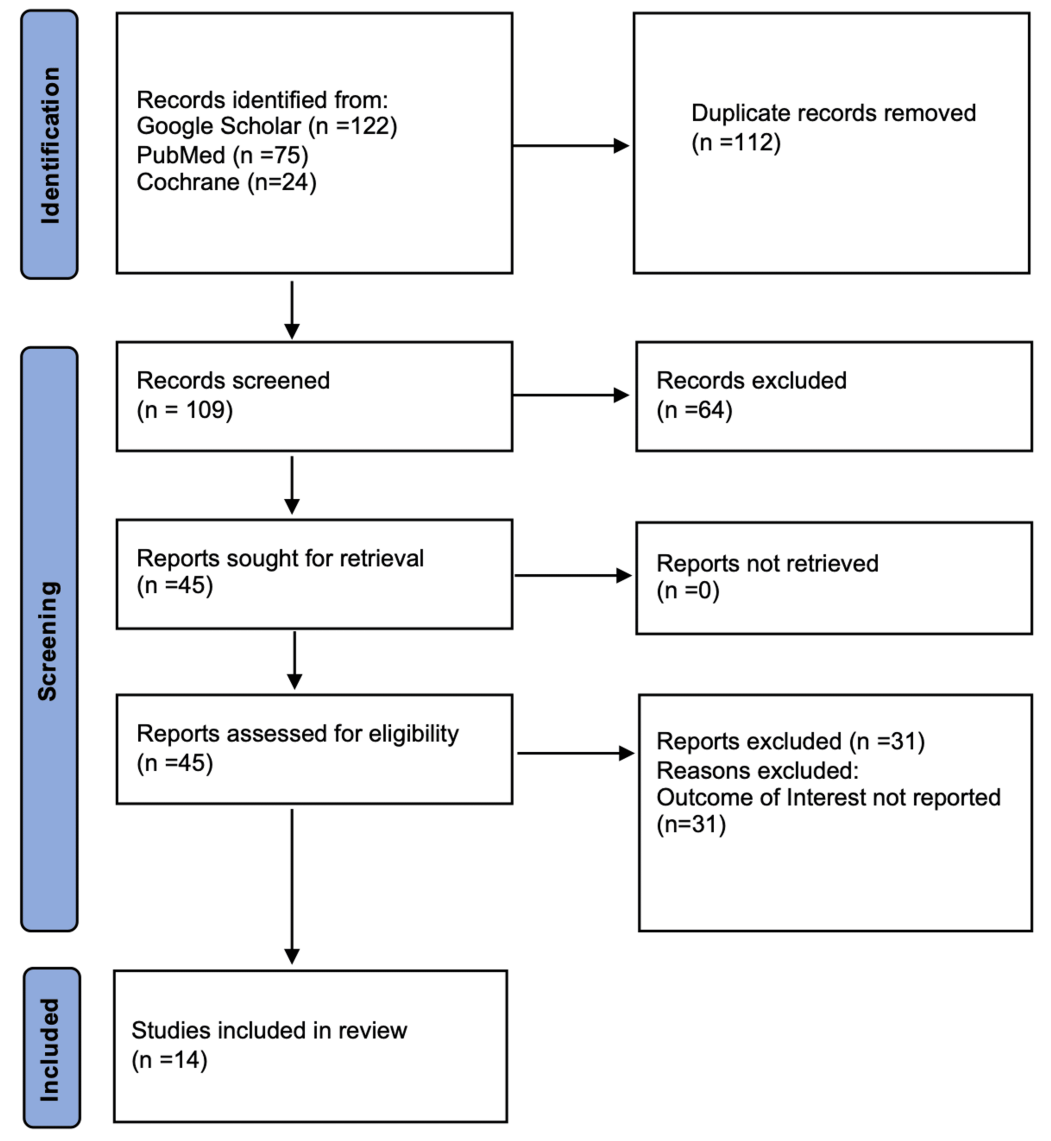
PRISMA flowchart

Fourteen studies were included in the analysis. The studies were published between 2009 and 2022, with their selection summarized in Figure 1. They were mostly from the USA (57.1%). The mean age of the patients was 42.58±15.12. 71% of the patients were female, with the majority receiving a hyaluronic acid (HA) filler injection (61.3%). The general study data is shown in Table 1.

**Table 1:** General study data HA, hyaluronic acid; AF, autologous fat; CaHa, calcium hydroxyapatite; PLLA, poly-L-lactic acid

Study	Region	Gender	Injected Substance	Management of Vascular Occlusion
Ansari et al., 2019 [12]	USA	1 Female	HA	Aspirin and prednisolone
Jolly et al., 2021 [13]	UK	1 Female	HA	Iopidine 1%, Hyaluronidase 1500 IU
Carle et al., 2014 [14]	USA	1 Male, 2 Females	HA, AF, Collagen	AC paracentesis
Chou et al., 2015 [15]	China	1 Female	CaHA	Alprostadil, Dextran, and high-pressure oxygen
Soares et al., 2023 [7]	USA	1 Female	CaHa	Morphine, Dexamethasone, Ketorolac
Georgescu et al., 2009 [16]	USA	1 Male, 1 Female	CaHa	Medrol DosePak, Nitroglycerin, and microdermabrasion
Sung et al., 2010 [17]	USA	1 Male	CaHa	Corticosteroids
Roberts et al., 2012 [18]	USA	1 Male	PLLA	Not mentioned
Kassir et al., 2011 [19]	USA	1 Male	HA	Massage, Mupirocin, Clindamycin, and Rocephin with silicone gel sheeting
Grunebaum et al., 2009 [20]	USA	1 Male, 2 Females	HA	Bacitracin, Debridement, Nitropaste, Hyaluronidase, Triamcinolone, and oral antibiotics
Pan et al., 2021 [21]	China	1 Female	HA	Massage, Hyaluronidase, and hyperbaric oxygen therapy
Beleznay et al., 2014 [22]	Canada	2 Males, 6 Females	CaHa, HA	Massage, warm compresses, nitroglycerin paste, hyaluronidase, prednisone, and aspirin
Thanasarnaksorn et al., 2018 [10]	Thailand	1 Male, 5 Females	HA	Carbogen, nitroglycerin, massage and Hyaluronidase with hyperbaric oxygen therapy
Cassiano et al., 2020 [23]	Brazil	1 Female	HA	Hyaluronidase and Prednisolone

The risk of bias assessment was done using the Joanna Briggs Institute Checklist for Case Reports [11] and is summarized in Table 2. Eight questions ranging from patient demographics to takeaway lessons assess the robustness, generalizability, and overall applicability of case reports in this critical appraisal tool.

**Table 2:** Critical appraisal of case reports using the Joanna Briggs Checklist

Study	Q1	Q2	Q3	Q4	Q5	Q6	Q7	Q8	Overall Appraisal
Ansari et al., 2019 [12]	Yes	Yes	Yes	Yes	Yes	Yes	Yes	Unclear	Include
Jolly et al., 2021 [13]	Yes	Yes	Yes	Unclear	Yes	Yes	Yes	No	Include
Carle et al., 2014 [14]	Yes	Yes	Yes	Yes	Yes	Yes	Yes	Unclear	Include
Chou et al., 2015 [15]	Yes	Yes	Yes	Yes	Yes	Yes	Yes	Unclear	Include
Soares et al., 2023 [7]	Yes	Yes	Yes	Yes	Yes	Yes	Yes	Yes	Include
Georgescu et al., 2009 [16]	Yes	Yes	Yes	No	Yes	Yes	Yes	Unclear	Include
Sung et al., 2010 [17]	Yes	Yes	Yes	Yes	Yes	Yes	Yes	No	Include
Roberts et al., 2012 [18]	Yes	Yes	No	Yes	Yes	Yes	Yes	Unclear	Include
Kassir et al., 2011 [19]	Yes	Yes	No	Yes	Yes	Yes	Yes	Yes	Include
Grunebaum et al., 2009 [20]	Yes	Yes	Yes	No	Yes	Yes	Yes	Unclear	Include
Pan et al., 2021 [21]	Yes	No	Yes	Yes	Yes	Yes	Yes	Yes	Include
Beleznay et al., 2014 [22]	Yes	Yes	Yes	No	Yes	Yes	Yes	Yes	Include
Thanasarnaksorn et al., 2018 [10]	Yes	No	Yes	Yes	Yes	Yes	Yes	Yes	Include
Cassiano et al., 2020 [23]	Yes	Yes	Yes	Yes	Yes	Yes	Yes	Yes	Include

The heterogeneity of the dataset further impacts the generalizability of findings. Variability in study methodologies, patient demographics, filler types, and management protocols complicates the interpretation of pooled data. For instance, the inconsistency in defining vascular occlusions and reporting recovery outcomes hampers cross-study comparisons. There is a clear need for standardized diagnostic criteria and reporting frameworks to improve data reliability and facilitate more comprehensive analyses in future research. Future research should focus on large-scale, prospective studies to validate these findings and refine management protocols. Additionally, developing a universally accepted risk stratification tool, such as the proposed morbidity risk assessment scale, could guide clinical decision-making and improve patient outcomes.

Risk Factor Analysis

Various risk factors for recovery status were accumulated after a thorough literature review. These included gender, injected substance, injection site, vessel involved, and time to presentation. The distribution of cases over these variables is shown in Table 3.

**Table 3:** Risk factor analysis with pooled OR HA, hyaluronic acid; AF, autologous fat; CaHa, calcium hydroxyapatite; PLLA, poly-L-lactic acid; OR, odds ratio

Variable		No Improvement (n=4)	Partial/Total Recovery (n=27)	OR (p-value)
Gender	Male	1	8	0.036 (0.8)
	Female	3	19	
Injected substance	HA	3	16	6.64 (0.21)
	CaHa	0	9	
	Others	1	2	
Injection site	Glabella	1	3	1.48 (0.24)
	Forehead	2	5	
	Nose	0	7	
	Periorbital	0	2	
	Cheek	1	3	
	Nasolabial fold	0	7	
Vessel involved	Ophthalmic artery	1	4	9.67 (0.02)
	Central retinal artery	0	6	
	Branch retinal artery	2	0	
	Facial artery	0	6	
	Angular artery	0	2	
	Multiple facial vessels	0	4	
	Others	1	5	
Time to onset/presentation	Immediately	2	11	3.13 (0.42)
	Less than 1 day	0	6	
	1 to 5 days	1	9	
	More than 5 days	1	1	

Eighteen cases (58.06%) showed complete recovery of functional impairment in the form of visual loss, ophthalmoplegia, or pain. Four cases (12.9%) showed no recovery after extensive therapy and remained permanently impaired in the form of non-aesthetic scar, permanent vision loss, or ptosis, among others. The primary association observed for partial or complete recovery was with the vessels involved; lesions affecting minor arteries (categorized as "others"), such as the supraorbital, supratrochlear, or infraorbital branch of the maxillary artery, demonstrated marginally better recovery outcomes than those involving other vessels (OR 9.67, p=0.02). Hyaluronidase was administered in all cases where complications arose due to HA fillers and was associated with an 84.2% rate of partial or total recovery.

Vascular Occlusion Morbidity Risk Assessment Tool

Weighted points were assigned to each risk factor based on the pooled ORs, forming an assessment scale described here. With a maximum total score of 14, a score greater than 9 indicated severe morbidity, often refractory to treatment. Risk categories were defined as follows: low-risk patients had a score of less than four, moderate-risk patients scored between four and nine, and high-risk patients had a score greater than nine (Table 4).

**Table 4:** Morbidity risk assessment tool HA, hyaluronic acid; AF, autologous fat; CaHa, calcium hydroxyapatite

Risk Factor		Point(s)
Gender	Female	2
	Male	1
Injected substance	HA	3
	CaHa	2
	Others	1
Injection site	Glabella/periorbital	3
	Forehead/nose	2
	Cheek	1
	Nasolabial fold	0
Vessel involved	Ophthalmic artery	4
	Central retinal artery	3
	Branch retinal artery	2
	Facial/angular artery	1
	Others	0
Time to onset/presentation	More than 5 days	2
	Less than 5 days	1
	Immediately	0

Clinical Implication

This tool offers significant clinical implications, promoting individualized patient care by identifying those requiring meticulous procedural adjustments or close post-procedural monitoring. For high-risk patients, practitioners can implement advanced injection techniques, such as using cannulas in high-risk areas or opting for safer filler types. Furthermore, it encourages early recognition of vascular compromise, facilitating timely interventions to prevent long-term complications. Integrating this tool into routine practice enhances safety and decision-making in aesthetic medicine, especially in high-stakes scenarios. The results of applying the morbidity risk assessment tool to our patient population are presented in Table 5.

**Table 5:** Risk assessment of the patient population

Case	Gender	Substance	Injection Site	Vessel	Onset	Total Points	Risk Category
Grunebaum et al., 2009 [20]	Female (2)	HA (3)	Nose (2)	Facial artery (1)	Immediately (0)	8	Moderate risk
Grunebaum et al., 2009 [20]	Male (1)	HA (3)	Nasolabial fold (0)	Facial artery (1)	<5 days (1)	6	Moderate risk
Pan et al., 2021 [21]	Female (2)	HA (3)	Forehead (2)	Central retinal artery (3)	<5 days (1)	11	High risk
Beleznay et al., 2014 [22]	Female (2)	CaHa (2)	Nasolabial fold (0)	Multiple facial vessels (1)	>5 days (2)	7	Moderate risk
Beleznay et al., 2014 [22]	Male (1)	HA (3)	Cheek (1)	Multiple facial vessels (1)	<5 days (1)	7	Moderate risk
Beleznay et al., 2014 [22]	Female (2)	CaHa (2)	Nose (2)	Angular artery (1)	<5 days (1)	8	Moderate risk
Beleznay et al., 2014 [22]	Female (2)	HA (3)	Nasolabial fold (0)	Facial artery (1)	<5 days (1)	7	Moderate risk
Beleznay et al., 2014 [22]	Female (2)	HA (3)	Nose (2)	Multiple facial vessels (1)	>5 days (2)	10	High risk
Beleznay et al., 2014 [22]	Female (2)	HA (3)	Nasolabial fold (0)	Facial artery (1)	<5 days (1)	7	Moderate risk
Beleznay et al., 2014 [22]	Male (1)	CaHa (2)	Glabella (3)	Supraorbital artery (1)	<5 days (1)	8	Moderate risk
Beleznay et al., 2014 [22]	Female (2)	CaHa (2)	Nasolabial fold (0)	Facial artery (1)	Immediately (0)	5	Moderate risk
Thanasarnaksorn et al., 2018 [10]	Female (2)	HA (3)	Cheek (1)	Ophthalmic artery (4)	Immediately (0)	10	High risk
Thanasarnaksorn et al., 2018 [10]	Female (2)	HA (3)	Nose (2)	Central retinal artery (3)	Immediately (0)	10	High risk
Thanasarnaksorn et al., 2018 [10]	Female (2)	HA (3)	Nose (2)	Central retinal artery (3)	Immediately (0)	10	High risk
Thanasarnaksorn et al., 2018 [10]	Male (1)	HA (3)	Nose (2)	Central retinal artery (3)	Immediately (0)	9	Moderate risk
Thanasarnaksorn et al., 2018 [10]	Female (2)	HA (3)	Forehead (2)	Ophthalmic artery (4)	Immediately (0)	11	High risk
Thanasarnaksorn et al., 2018 [10]	Female (2)	HA (3)	Temporal area (1)	Central retinal artery (3)	Immediately (0)	9	Moderate risk
Cassiano et al., 2020 [23]	Female (2)	HA (3)	Forehead (2)	Supratrochlear artery (1)	<5 days (1)	9	Moderate risk

Discussion

**Figure 2:**
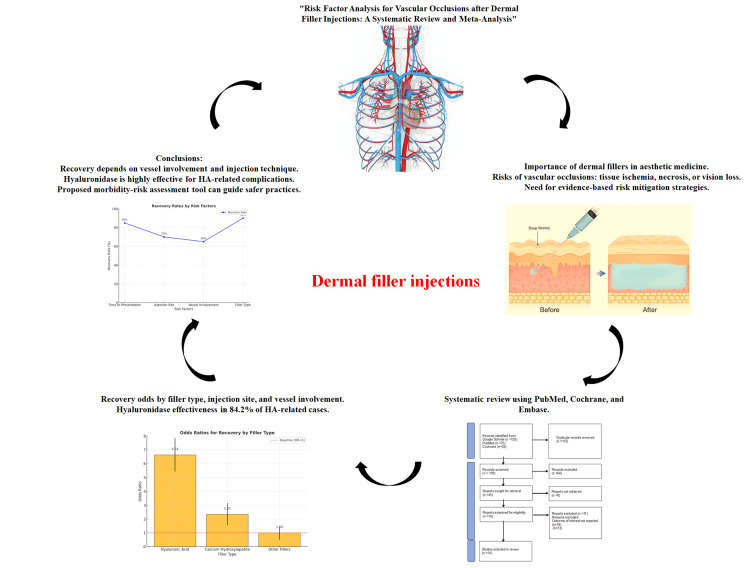
Graphical abstract of the study Image credits: Authors

This study looked into the vascular occlusions related to using any aesthetic filler injection while addressing possible risk factors, management, and clinical outcomes (a graphical abstract of the study is shown in Figure 2). Its results recommend that a customized approach toward aesthetic medicine would enhance patient safety and give an optimized effect in the treatments offered [24]. The predominance of vascular occlusions in the female population studied was 71%, corresponding with the demographic trend that most patients who undergo cosmetic procedures in facial enhancement are women [25]. This gender disparity, however, might also reflect anatomical blockages or physiological differences that predispose female patients to such complications. HA has been implicated as a filler in almost 61.3 percent of the cases analyzed. This may well be attributed to the general inclination of individuals for the use of such fillers, given that they are biocompatible as well as reversible by hyaluronidase, and not due to a tendency to be more naturally occlusive with blood vessels in comparison with other fillers such as CaHa or PLLA.

The location in which the filler is injected is regarded as the most critical parameter for assessing vascular risk. The occlusive events have occurred most frequently in high-risk anatomic zones such as the glabella, nose, and nasolabial folds. Rich vascular networks, such as the ophthalmic and angular arteries, have direct connections to the retinal circulation, making these areas particularly susceptible to occlusions and increasing the severity of resulting complications, such as blindness and extensive tissue necrosis [26]. Comparatively, higher site areas like the lateral cheek and jawline have been noted to significantly decrease occlusion incidences, indicating that risk evaluation should form part of a procedure's site-specific component. Occlusions involving minor arteries, such as the supraorbital, supratrochlear, and infraorbital branches, demonstrated better recovery odds (OR 9.67, p=0.02), likely due to their smaller caliber and limited vascular territory. This is why there is a need for anatomical precision during injections, particularly in high-risk zones.

The time taken to present is critical to the clinical outcome of the patient. Immediate recognition of vascular compromise within hours of injection is associated with very good recovery, while recognition of such events much later (greater than five days) is associated with poor prognosis with irreversible tissue loss and possible vision loss. It emphasizes the need for better awareness among both the practitioners and patients regarding the early signs of vascular occlusion (blanching, pain, and livedo reticularis) as warning signals. The monitoring of patients in these intervention procedures should be in real-time because it is important to document early signs of intravascular injection, including resistance to injection and immediate blanching. Such events require immediate cessation and corrective action.

Managing vascular occlusions was primarily dependent on hyaluronidase treatment, typically indicated for cases involving HA fillers. Administration of targeted high-dose hyaluronidase injections was established to yield an 84.2% chance of partial or complete recovery, reaffirming it as the first-line treatment. Augmentative treatment options such as nitroglycerin paste, aspirin, corticosteroids, and hyperbaric oxygen therapy had, however, complemented recovery in some cases.

The inconsistency across studies throws a glaring light on an urgent need to develop treatment protocols and guidelines for filler-induced vascular events [27,28].

The expertise of the practitioner was another significant factor in avoiding complications owing to the possibility of high incidence of vascular occlusions caused by faulty techniques used. The safest measures hence recommended included the use of blunt-tipped cannulae rather than sharp needles in injection high-risk areas, slow retrograde injections, and repeated aspiration before injection to prevent intravascular placements. Similarly, before any procedure, vascular mapping using imaging modalities such as ultrasound can also give a proactive approach to high-risk anatomical variations and reduce complication rates. Such practice of case selection will continue being competent in risk mitigation and at promoting individual evaluations for detection of candidates at an even higher risk of developing vascular occlusions. Besides the anatomy, preexisting vascular conditions, history of thromboembolic events, and previous adverse reactions to dermal fillers should inform decision-making. This suggested tool for vascular occlusion morbidity-risk assessment could become a promising framework that one will use in stratifying low, moderate, and high-risk patients so that they can have their own adjusted and personalized procedural and post-treatment monitoring.

There is a clear need for standardized diagnostic criteria and reporting frameworks to improve data reliability and facilitate more comprehensive analyses in future research. Future research should focus its efforts on large-scale, prospective studies, which will prove to be strategic in validating these observations and refining evidence-based management protocols. The standardization of risk stratification tools and the consensus-derived treatment algorithms would go a long way toward standardizing safety measures and thereby enhancing patient outcomes in aesthetic medicine.

Developing and adhering to evidence-based guidelines for managing vascular occlusions can reduce variability in outcomes. A unified protocol that incorporates hyaluronidase dosing, timing, and adjunctive therapies would be particularly beneficial. This study reinforces that prompt recognition and intervention are critical for favorable outcomes. Occlusions presenting within one day were associated with higher recovery rates, while delays beyond five days correlated with poor outcomes. Standardized treatment protocols, including immediate administration of hyaluronidase for HA fillers and adjunctive therapies, should be widely adopted to ensure consistency in managing complications.

This study looks into the multifaceted nature of vascular occlusion and takes into consideration the anatomical risk factors, procedural techniques, and timely interventions. These findings highlight the importance of practitioner training in specific assessments of individual patient risk, along with the management protocol for trying to reduce the occurrence of complications. The use of hyaluronidase remains a cornerstone in treating occlusions with HA, but an integrated approach employing different adjunct therapies and different procedural safeguards will ensure maximum healing. While aesthetic medicine is constantly evolving, research and clinical compliance will remain important to the safety and efficacy of dermal filler procedures.

However, while this study presents valuable findings (Table 6), there are limitations to its interpretation. The dependence on case reports and retrospective data brings with it all of the biases inherent in such studies, including variability in reporting standards and a disproportionate emphasis placed on severe or otherwise atypical events. This will therefore lead to an underreporting of milder or transient vascular occlusions, although the general trend may be skewed toward more dramatic presentations. In addition, the variability in the methodology used in determining the studies, the patients uninvolved, and the different filler types used complicate comparison across studies and render the findings less generalized. The heterogeneity of the dataset further impacts the generalizability of findings. Variability in study methodologies, patient demographics, filler types, and management protocols complicates the interpretation of pooled data. For instance, the inconsistency in defining vascular occlusions and reporting recovery outcomes hampers cross-study comparisons.

**Table 6:** Summary of findings HA, hyaluronic acid

Finding Area	Summary
Most common filler material	HA (61.3%) was most commonly implicated
Recovery with hyaluronidase	84.2% of HA-related occlusions showed partial or complete recovery with hyaluronidase
Vessel involvement and prognosis	Minor artery involvement showed better recovery (OR 9.67, p=0.02)
High-risk injection sites	Glabella, nose, and nasolabial folds were most associated with severe complications
Gender distribution	71% of affected patients were female
Impact of time to presentation	Early presentation (immediate or <1 day) correlated with better recovery
Overall recovery outcomes	58% full recovery; 12.9% showed no recovery and had permanent deficits
Risk stratification tool	Tool developed to stratify patients into low, moderate, and high-risk groups

## Conclusions

This meta-analysis identifies key procedural and anatomical risk factors contributing to vascular occlusion following dermal filler injections. Findings underscore the importance of proper injection technique, detailed anatomical knowledge, and prompt recognition of vascular compromise to reduce the risk of serious complications such as tissue necrosis or vision loss. High-risk facial regions, particularly the glabella, nose, and nasolabial folds, were most commonly associated with adverse outcomes, reinforcing the need for advanced practitioner training and refined injection approaches. The study also highlights the vital role of hyaluronidase in treating complications related to HA fillers, with a high proportion of cases showing partial or complete recovery following timely administration.

Nonetheless, the conclusions drawn are limited by the retrospective nature of the data, variability in case reporting, and a lack of uniform diagnostic criteria across studies. The small sample size and inconsistency in methodology prevented detailed subgroup analyses on the impact of factors like demographics, comorbidities, and specific injection techniques. These limitations highlight the urgent need for standardized definitions, reporting frameworks, and clinical protocols to ensure consistency in both research and practice. Future research should prioritize multicenter, prospective studies with larger and more diverse patient populations to validate these findings and refine best practices. The development of comprehensive, evidence-based guidelines for procedural safety, anatomical risk mapping, and emergency intervention protocols is essential. Moreover, incorporating patient-specific factors, such as individual vascular anatomy and medical history, into pre-procedural planning can further personalize care and improve outcomes. These efforts will be instrumental in enhancing safety standards and advancing the quality of care in aesthetic medicine.

## Appendices

**Table 7:** Databases

Database	Search Strategy
PubMed	("vascular occlusion"[MeSH Terms] OR "vascular occlusion"[Title/Abstract] OR "arterial occlusion"[Title/Abstract] OR "venous occlusion"[Title/Abstract]) AND ("dermal fillers"[MeSH Terms] OR "dermal fillers"[Title/Abstract] OR "injectable fillers"[Title/Abstract] OR "soft tissue augmentation"[Title/Abstract]) AND ("risk factors"[MeSH Terms] OR "risk factors"[Title/Abstract] OR "complications"[Title/Abstract] OR "adverse events"[Title/Abstract]) AND ("case-control studies"[MeSH Terms] OR "case-control"[Title/Abstract] OR "cohort studies"[MeSH Terms] OR "cohort"[Title/Abstract] OR "case series"[Title/Abstract] OR "retrospective"[Title/Abstract] OR "prospective"[Title/Abstract])
Google Scholar	"vascular occlusion" OR "arterial occlusion" OR "venous occlusion" AND "dermal fillers" OR "injectable fillers" OR "soft tissue augmentation" AND "risk factors" OR "complications" OR "adverse events" AND ("case-control" OR "cohort" OR "case series" OR "retrospective" OR "prospective")
Cochrane	("vascular occlusion" OR "arterial occlusion" OR "venous occlusion") AND ("dermal fillers" OR "injectable fillers" OR "soft tissue augmentation") AND ("risk factors" OR "complications" OR "adverse events") AND ("case-control" OR "cohort" OR "case series" OR "retrospective" OR "prospective")
